# Emerging Trends in HIV-1 Sub-Subtype A6 in Belgium: Transmission Dynamics, Drug Resistance, and Subtyping Tool Evaluation

**DOI:** 10.3390/v18050554

**Published:** 2026-05-12

**Authors:** Virginie Mortier, Laurent Debaisieux, Deborah De Geyter, Marie-Luce Delforge, Melissa Depypere, Géraldine Dessilly, Benoît Kabamba-Mukadi, Khalid El Moussaoui, Samy Mzougui, Ben Serrien, Karolien Stoffels, Dominique Van Beckhoven, Ellen Van Cutsem, Dorien Van den Bossche, Sigi Van den Wijngaert, Fien Vanroye, Elizaveta Padalko, Chris Verhofstede, Kristel Van Laethem

**Affiliations:** 1 AIDS Reference Laboratory, Department of Diagnostic Sciences, Ghent University, 9000 Ghent, Belgium; elizaveta.padalko@ugent.be (E.P.); chris.verhofstede@ugent.be (C.V.); 2 AIDS Reference Laboratory, Université Libre de Bruxelles (ULB), Hôpital Universitaire de Bruxelles (H.U.B), CUB Hôpital Erasme, 1070 Brussels, Belgium; laurent.debaisieux@hubruxelles.be (L.D.); marie-luce.delforge@hubruxelles.be (M.-L.D.); 3 AIDS Reference Laboratory, Vrije Universiteit Brussel VUB, 1090 Brussels, Belgium; deborah.degeyter@uzbrussel.be (D.D.G.); ellen.vancutsem@uzbrussel.be (E.V.C.); 4 Department of Microbiology, Immunology and Transplantation, KU Leuven, 3000 Leuven, Belgium; melissa.depypere@uzleuven.be (M.D.); kristel.vanlaethem@uzleuven.be (K.V.L.); 5 AIDS Reference Laboratory, University Hospitals Leuven, 3000 Leuven, Belgium; 6 AIDS Reference Laboratory, Medical Microbiology Unit, Université Catholique de Louvain, 1200 Brussels, Belgium; geraldine.dessilly@uclouvain.be (G.D.); benoit.kabamba@saintluc.uclouvain.be (B.K.-M.); 7 AIDS Reference Laboratory, University Hospital of Liege, 4000 Liege, Belgium; kelmoussaoui@chuliege.be (K.E.M.); samy.mzougui@chuliege.be (S.M.); 8 Department of Epidemiology and Public Health, Sciensano, 1050 Brussels, Belgium; ben.serrien@sciensano.be (B.S.); dominique.vanbeckhoven@sciensano.be (D.V.B.); 9 AIDS Reference Laboratory, Centre Hospitalier Universitaire St. Pierre, 1000 Brussels, Belgium; karolien.stoffels@lhub-ulb.be (K.S.); sigi.vandenwijngaert@lhub-ulb.be (S.V.d.W.); 10 AIDS Reference Laboratory, Department of Clinical Sciences, Institute of Tropical Medicine, 2000 Antwerp, Belgium; dvandenbossche@itg.be (D.V.d.B.); fvanroye@itg.be (F.V.); 11 Clinical Reference Laboratory, Department of Clinical Sciences, Institute of Tropical Medicine, 2000 Antwerp, Belgium; 12 Department of Medical Microbiology, Ghent University Hospital, 9000 Ghent, Belgium

**Keywords:** human immunodeficiency virus, sub-subtype A6, transmission dynamics, genotypic drug resistance, subtyping tools

## Abstract

The international spread of HIV-1 sub-subtype A6 raises concerns due to its association with contraindications for long-acting injectable formulations of cabotegravir (LA-CAB) and rilpivirine (LA-RPV). This study investigated its increasing proportion in Belgium, assessing transmission dynamics and potential migration links. Additionally, genotypic drug resistance in the Belgian HIV-1 sub-subtype A6 population were analyzed and four automatic subtyping tools were compared. A dataset of 4764 HIV-1 protease and reverse transcriptase (RT) sequences from newly diagnosed, treatment-naïve individuals in Belgium (2013–2022) was analyzed. A combination of phylogenetic analysis and online subtyping tools identified 136 sub-subtype A6 sequences. The increase in the proportion of HIV-1 sub-subtype A6 observed in Belgium since 2020 reflects changing transmission patterns, especially among Belgium-born men having sex with men, and cannot be solely linked to the recent influx of Ukrainian migrants. Of these sub-subtype A6 sequences, less than 10% showed LA-CAB + LA-RPV resistance, mainly due to E138A within RT. HIVdb and ANRS reliably assessed resistance in this therapy-naïve cohort, and HIVdb, COMET, and SmartGene^®^ produced concordant subtyping results. While algorithm choice has little impact at low resistance prevalence, further research is necessary and HIVdb and ANRS remain more suitable for ongoing clinical and research use.

## 1. Introduction

Human Immunodeficiency Virus type 1 (HIV-1) remains a significant global public health concern, where the prevalence and distribution of subtypes and recombinants play a crucial role in shaping the epidemiological landscape [1]. Human migration and increased mobility have been identified as key drivers of HIV-1 spread across nations, thereby intensifying the complexity of viral genetic diversity with a potential impact on the effectiveness of testing, prevention and treatment strategies.

Of particular concern is the international expansion of a lineage within HIV-1 subtype A, which has garnered attention due to its association with contraindications for a long-acting injectable formulation of cabotegravir and rilpivirine (LA-CAB + LA-RPV) [2]. Cabotegravir, a second generation integrase (IN) strand transfer inhibitor (INSTI), and rilpivirine, a non-nucleoside reverse transcriptase (RT) inhibitor (NNRTI), are increasingly prescribed as treatment or as pre-exposure prophylaxis (PrEP) for a subset of patients preferring long-acting injections over daily oral tablets [3,4,5,6,7]. The combination LA-CAB + LA-RPV demonstrated a very high efficacy in the FLAIR, ATLAS and ATLAS-2M phase III studies. However, a post hoc multivariate analysis identified three factors associated with confirmed virological failure when two of the three are present, namely: presence of RPV resistance-associated mutations (RAMs), infection with HIV-1 sub-subtype A6 and/or a baseline body mass index (BMI) of at least 30 kg/m^2^ [8]. Due to some classification difficulties within HIV-1 subtype A, especially with automatic subtyping tools commonly used in routine clinical practice, the variants found in patients who, in the phase III studies, discontinued treatment because of lack of virologic activity, were originally misclassified as sub-subtype A1 instead of A6 [3,4,5].

Already in 2018, Désiré et al. (2018) [9] proposed an updated and more accurate nomenclature of HIV-1 subtype A with its division into six sub-subtypes, called A1, A2, A3, A4, A6 and A7. More recently, a seventh HIV-1 sub-subtype A8 was described in Cabo Verde [10]. HIV-1 subtype A variants are predominantly found in East and Central Africa, Eastern Europe and Central Asia [1], of which only sub-subtype A1 and A6 have established major epidemics. HIV-1 sub-subtype A1 is highly prevalent in East Africa, Greece and Cyprus [11,12], whereas sub-subtype A6 is predominantly found in the Eastern European Region (EER). Bayesian phylogeographic analyses support the introduction of sub-subtype A6 from the Democratic Republic of Congo (DRC) into the EER, locating this introduction in Odessa [13]. The Ukrainian city of Odessa is situated at the Black Sea and has a major seaport which was an important trade center and naval base in the former Soviet Union (FSU). Initially, this viral variant was locally circulating at low transmission rates among heterosexuals (HET), but after a single point introduction among intravenous drug users (IDUs) it was spread explosively to other Ukrainian cities and other countries previously belonging to the FSU. The geographical spread of sub-subtype A6 is increasing in Central and Western Europe, especially in Poland where Ukrainian citizens became the dominant group of migrants since the start of the Russo-Ukrainian conflict in 2014 and where more than 6 million Ukrainians sought refuge since the total invasion in 2022 [2,14,15].

Since the eighties, HIV-1 subtype A has been one of the most prevalent non-B subtypes in Belgium, mainly detected among HET and reflecting the viral genetic diversity observed in Central Africa [16,17]. Recently, a substantial increase in HIV-1 subtype A infections has been observed among newly diagnosed and treatment-naïve HIV-1 patients in Belgium [18]. This observation prompted a comprehensive investigation of HIV-1 subtype A infections to delineate the distinct sub-subtypes within our epidemic and to ascertain the factors associated with this increased prevalence. Specifically, our study aimed to determine whether the increase could be attributed to sub-subtype A6 and whether it was solely linked to migratory patterns or also influenced by local transmission dynamics among specific risk groups. Additionally, a detailed investigation of the genotypic drug resistance profile was performed.

## 2. Materials and Methods

### 2.1. Viral Sequences and Associated Information from HIV-1 Epidemic in Belgium

A dataset of 4764 HIV-1 protease and reverse transcriptase (PR-RT) sequences was collected from people newly diagnosed with HIV-1 in Belgium between 2013 and 2022, who were treatment naive and received baseline genotyping as part of routine clinical care at one of the seven Belgian AIDS Reference Laboratories (ARL). In 2019, baseline genotyping was extended to the HIV-1 IN region, enabling the inclusion of an additional 1674 treatment-naïve IN sequences from 1704 newly diagnosed HIV-1 infections since 2019 (98.2%). Sequences were generated from blood plasma samples collected as soon as possible after diagnosis using either an in-house method, ViroSeq HIV-1 Genotyping System (Abbott Molecular, Des Plaines, IL, USA), TRUGENE HIV-1 Genotyping Assay (Siemens Diagnostics, Tarrytown, NY, USA), or Sentosa SQ HIV-1 Genotyping Assay (Vela Diagnostics, Hamburg, Germany) with a 5% cut-off or a 20% cut-off if the viral load is below 1000 copies/mL.

Socio-demographic and laboratory information on new HIV-1 diagnoses were collected from healthcare providers by the ARL within the framework of mandatory and pseudonymized reporting to the Belgian Institute for Public Health (Sciensano, Brussels). Sciensano coordinates the quality control process between data providers and data analysts who are jointly responsible for HIV/AIDS surveillance in Belgium. In this study, pseudonymized identifiers were used to assemble the deduplicated sequences annotated with verified data on sex, year of birth, year of diagnosis, country of birth and risk category. The following countries were defined by ECDC to be part of the Eastern European Region (EER): Armenia, Azerbaijan, Belarus, Estonia, Georgia, Kazakhstan, Kyrgyzstan, Latvia, Lithuania, Republic of Moldova, Russian Federation, Tajikistan, Turkmenistan, Ukraine and Uzbekistan [19].

### 2.2. HIV-1 Subtyping, Phylogenetic Analysis and Transmission Cluster Analysis

HIV-1 subtype classification was initially performed on all 4764 PR-RT sequences using the online automated subtyping tool from Stanford HIV Drug Resistance Database version 9.6 (HIVdb v9.6) [20,21]. Only sequences that were entirely classified as subtype A over the full length of the sequenced fragment were retained and additional subtyping of these 478 sequences was performed using the COMET HIV-1 v2.4 [22] and Rega v3.46 [23] algorithms. Recombinant sequences, unassigned sequences and sequences marked as ‘check the report’ were removed. The remaining 427 sequences were aligned in Bioedit v7.0.5 [24] and manual phylogenetic analysis resulted in the identification of 136 sequences of sub-subtype A6 (Figures S1 and S2). For this purpose, 13 HIV-1 sub-subtype A6 references (EU861977, FJ864679, JQ2928976, JX500694, KF716491, KU749406, KY658681, MF109697, MG902951, MH330381, MN703139, MT370007, MZ427743) and 35 references of other HIV-1 A subtypes (A1: AF004855, AF069670, AF484509, U15590, MN650447, AB485632, MN650486, OR508687, JF683783; A2: AF286240, AF286237, AF286238, MH705163, GU201516, DQ136736; A3: FJ842349, AY521630, AY521629, GU367484, AY521631, MF109645; A4: AM000055, AM041020, AM000053, AM000054; A7: MH078558, KX389608, KX389622, FJ388928, GU207033; A8: AY165240, MW353966, MW353970, MW353969, MW353967) from the Los Alamos HIV Sequence Database (LANL) [25] were aligned with our study sequences using BioEdit v7.0.5. The part of the PR-RT and IN sequence covered by all the amplification and sequencing methods used was retained. This resulted in phylogenetic analysis performed on a PR-RT fragment of 870 nucleotides long, spanning amino acids 9 to 99 in PR and amino acids 41 to 239 in RT and an IN fragment of 867 nucleotides long, spanning amino acids 1 to 289 in IN.

The maximum likelihood approach implemented in PhyML v3.0 with automatic selection of the best-fit, evolutionary model of deoxyribonucleic acid substitution was used for phylogenetic analysis [26] and iTOL v6.7.6 was used for tree visualization (https://itol.embl.de/). For cluster identification, ClusterPicker v1.2 with a bootstrap value of ≥0.90 and a genetic distance threshold of 2.5% was applied [27]. Clusters were defined as groups of three or more sequences.

As HIV-1 sub-subtype A6 was one of the baseline risk factors for reduced susceptibility to LA-CAB + LA-RPV, subtype classification was also performed with the integrated database network system (IDNS) SmartGene^®^ v3_13_0(r32707) (Lausanne, Switzerland) used by the Belgian ARL for genotypic drug resistance interpretation in a routine setting. SmartGene^®^ is the only algorithm used in this analysis that performs subtyping independently for both PR-RT and IN regions.

### 2.3. Assessment of Genotypic Baseline Risk Factors for RPV and CAB Failure

PR-RT and IN sequences were assessed for RPV and INSTI resistance mutations according to the list of RAMs used within the ad hoc analysis of the phase III clinical studies [8]. For comparison, the genotypic susceptibility profiles for RPV and CAB were also assessed according to HIVdb v9.6 and ANRS v35, as these algorithms are used for routine clinical purposes in Belgium within the IDNS SmartGene^®^. HIVdb scores between 10 and 14 indicate potential low-level resistance (PLLR), while scores of 15 to 29 in HIVdb low-level resistance (LLR), scores of 30 to 59 intermediate resistance (IR) and a score of 60 high-level resistance (HLR). For comparison reasons, this original 5-level HIVdb resistance score was converted to the commonly used 3-level resistance score: susceptible (S; 0–14), intermediate resistant (IR; 15–59) and resistant (R; ≥60), and all mutations within the list of Cutrell et al. (2021) [8] were considered to confer resistance (R).

## 3. Results

### 3.1. Characteristics of People Living with HIV (PLWH) Infected with HIV-1 Sub-Subtype A6

Seven thousand one hundred and twenty-eight people were diagnosed with an HIV-1 infection between 2013 and 2022 in Belgium (Table 1). Four thousand seven hundred and sixty-four people were therapy-naïve and received a baseline genotypic drug resistance testing as part of their routine clinical care. Of them, one hundred and thirty-six treatment-naïve individuals were diagnosed with an HIV-1 sub-subtype A6 infection (136/4764; 2.9%). For a subset of these individuals (76/136; 55.9%) an IN sequence was available for subtyping and this analysis confirmed the A6 result. The median age of people newly diagnosed with an HIV-1 sub-subtype A6 infection was 36 years (IQR: 31–47) and the majority were male (77.9%). Data on the country of birth was available for 96 persons, revealing a distribution of 38.5% and 51.0% people born in Belgium and in the EER, respectively. In terms of disclosed risk category, data was available for 100 individuals and revealed that 50.0% were men who have sex with men (MSM), 40.0% were HET, and 10.0% were IDU.

**Figure 1:**
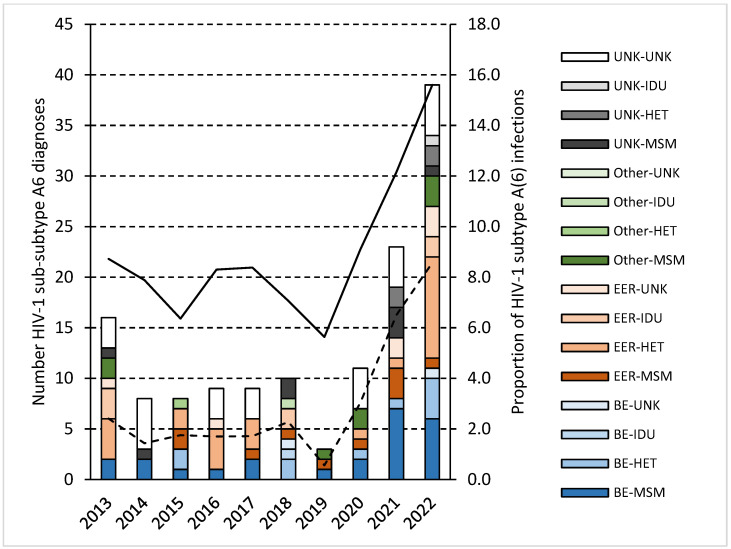
Proportion of HIV-1 pure subtype A (solid line) and sub-subtype A6 infections (dash line) among newly diagnosed, therapy-naïve individuals in Belgium (2013–2022) and number of HIV-1 sub-subtype A6 diagnoses according to country of birth and risk category (bars). BE, Belgium; EER, Eastern European Region; UNK, unknown; MSM, men having sex with men; HET, heterosexual; IDU, intravenous drug use.

A remarkable increase in diagnosis of people infected with HIV-1 sub-subtype A6 was observed since 2020: less than 10 in 2014–2019 and >10 from 2020 onwards to reach 39 in 2022 (Figure 1). The increase in diagnosis of sub-subtype A6 is attributable to infections among individuals born in Belgium or the EER and to both HET and MSM transmissions.

Phylogenetic analysis of all 136 PR-RT sequences revealed the presence of three clusters comprising 3, 5 and 21 individuals (Figures S1 and S2). The smallest cluster was composed of three MSM born in, respectively, Belgium, Ukraine and France, all three diagnosed in 2020. The clusters of 5 and 21 individuals were still expanding in 2022, suggesting recent local onward transmission. In the cluster comprising five individuals, two were born in Belgium and one in Brazil, four were MSM and four received a diagnosis in 2022, while one was diagnosed in 2018. Among the cluster of 21 individuals, 14 individuals were identified as born in Belgium and 2 in a Northern African country. Additionally, out of the 18 PLWH with known infection risk within this cluster, 15 were MSM and 3 individuals were HET. Timing of diagnosis for the individuals of this cluster were: 1 diagnosis in 2019, 3 in 2020, 11 in 2021 and 6 in 2022. Of note, none of the patients occurring in clusters are suspected to have been infected through IDU and only one patient was born in the EER.

### 3.2. Performance Evaluation of Automatic Subtyping Tools

Four hundred and twenty-seven sequences were identified as pure HIV-1 subtype A based upon a combined approach of three free online automated subtyping tools, i.e., Stanford HIV Drug Resistance Database version 9.6 (HIVdb v9.6) [20,21], COMET HIV-1 v2.4 [22] and Rega v3.46 [23], and manual phylogenetic analysis. The performance of these three online automated subtyping tools with respect to HIV-1 sub-subtype A6 classification, as well as of SmartGene^®^ (commercial software) that is used by the Belgian ARL in routine practice, was evaluated for this specific subset of sequences (Table 2). HIVdb accurately identified all A6 sub-subtype sequences as A6, but it also incorrectly classified 15 of the 291 non-sub-subtype A6 sequences as A6 (accuracy: 0.96, sensitivity: 1.00, specificity: 0.95). COMET showed a high accuracy rate, assigning 134 of the 136 sub-subtype A6 sequences to A6, and the remaining two as A1. Notably, COMET did not misidentify any non-sub-subtype A6 sequences as sub-subtype A6 (accuracy: 0.99, sensitivity: 0.98, specificity: 1.00). Rega can only discriminate between sub-subtype A1 and sub-subtype A2 among A subtypes. Hence, all 136 sub-subtype A6 sequences were misclassified as sub-subtype A1 (accuracy: 0.68, sensitivity: 0.00, specificity: 1.00). SmartGene’s^®^ tool accurately classified all sequences as sub-subtype A6 and as non-sub-subtype A6 (accuracy: 1.00, sensitivity: 1.00, specificity: 1.00). Of note, 19 of 291 non-sub-subtype A6 sequences were assigned to sub-subtype A6 in the protease region, but to another A-subtype in the reverse transcriptase region.

### 3.3. Frequency of RPV and CAB RAMs in Sequences of Sub-Subtype A6

Overall and according to the RAMs listed by Cutrell et al. (2021) [8], the presence of at least one RPV RAM was observed in 6.6% of the sequences (9/136), with E138A being the sole observed RAM (Table S1). This finding was corroborated by the ANRS algorithm, scoring variants with E138A as resistant. According to the HIVdb resistance algorithm, 8.8% of the sequences (12/136) displayed any RAM, encompassing E138A (6.6%), G190S (1.5%) and V179D (0.7%). However, variants containing E138A or G190S are considered to be intermediate resistant, and variants displaying V179D still susceptible. Of note, mutational combination patterns resulting in intermediate or resistant scores to RPV were not detected when taking into account the HIVdb v9.6 and ANRS v35 rules.

In the same vein, considering Cutrell et al. (2021) [8], the presence of at least one CAB RAM was observed in 98.7% (75/76) (Table S2). This was mainly due to the mutation L74I that was observed in 96.1% of the sequences, but also to the presence of L74M (2.6%), Y143C (1.3%) and G193E (3.9%). According to the HIVdb resistance algorithm, only 3.9% of the sequences (3/76) displayed one CAB RAM, as only L74M (2.6%) and Y143C (1.3%) were identified as CAB RAM by HIVdb. No CAB RAMs were identified by the ANRS algorithm. None of the variants were scored intermediate or resistant when taking into account the HIVdb v9.6 and ANRS v35 rules applying to single mutations or mutational combination patterns.

Importantly, if not taking into consideration the CAB RAM L74I, none of the sequences exhibited RAMs associated with resistance to both RPV and CAB.

## 4. Discussion

Our findings reveal the presence of HIV-1 sub-subtype A6 among newly diagnosed individuals in Belgium since 2013, with its proportion among diagnoses remaining initially relatively stable at levels below 10 cases per year for approximately seven years. However, a marked increase was observed from 2020 on, reaching a level of 39 cases in 2022. Initially, sub-subtype A6 was mainly, though not exclusively, identified in migrants. Since 2020, the sub-subtype A6 epidemic displays a dual dynamic, with singleton and pair infections observed among both HET of Belgian and EER descent, alongside active transmission clusters within the MSM population, with mainly people born in Belgium. No transmission clusters characterized by solely IDU or HET risk factors were detected, despite these being the dominant transmission routes in the EER [28]. The recent increase in diagnoses can hence not solely be attributed to the high migration rate of Ukrainians following the Russian invasion of Ukraine on 24 February 2022.

A similar observation was made for the HIV-1 sub-subtype F1 in Belgium, for which initially only three cases were observed between 2001 and 2003 and that subsequently exponentially spread among MSM to reach a prevalence of 11.1% among new HIV-1 infections in 2015 [29]. Early treatment efforts eventually successfully limited the broader dissemination of F1, but the strain remains prevalent within the MSM population up until now [18]. This is in agreement with other European studies where the increase in HIV-1 non-B subtypes is not driven by migration but by local transmission in mainly non-HET networks [30,31,32,33,34,35].

Due to the contraindications associated with LA-CAB + LA-RPV, HIV-1 sub-subtype classification within subtype A should be implemented in routine practice where subtyping is often based on rapid automated tools. The most commonly used tools are HIVdb, COMET and Rega. HIVdb is a similarity-based tool where the query sequence is aligned to a selection of reference HIV-1 subtype sequences and a subtype is assigned based upon percentage identity. A similar approach is used by the SmartGene^®^ tool that is routinely employed by the Belgian ARL. In contrast, COMET distinguishes itself from foregoing methods that incorporate more time-consuming alignments. Instead, it compares nucleotide frequencies between query and reference sequences, using a likelihood model to assign a subtype. Rega requires even more computing time as it is a phylogenetics-based tool that inherently is less reproducible than the above deterministic tools. It did not detect any of the sub-subtype A6 sequences. Very high sensitivities for HIV-1 sub-subtype A6 were observed for the other three tools. Successful classification of both sub-subtype A6 and non-sub-subtype A6 sequences were achieved using the SmartGene^®^ tool, provided that both the PR and RT sequences were taken into account. HIVdb accurately identified all sub-subtype A6 sequences as A6, but it also incorrectly classified 15 of the 291 non-sub-subtype A6 sequences as A6. This misclassification could potentially result in denying long-acting medications to individuals who otherwise might benefit from them. In contrast, COMET accurately identified all non-sub-subtype A6 sequences, avoiding any misclassification as A6. However, it missed two sub-subtype A6 sequences, classifying them as A1. This could potentially result in prescribing long-acting medication to individuals with contraindications. Nonetheless, it is important to emphasize that there are only recommendations to withhold long-acting treatments when at least two of three contraindications are present, being infection with a strain of sub-subtype A6, resistance to RPV, or a high BMI [8]. In conclusion, while phylogenetic analysis remains the most reliable method for subtyping, it is also the most labor-intensive and time-consuming approach. Consequently, alternative tools like COMET, HIVdb, and SmartGene^®^ offer practical options, despite the small risk of misinterpretation. This underscores that periodic updating with the latest and most relevant HIV-1 reference sequences is fundamental for all subtyping methods, whether they are automatic tools or methods using manual phylogenetic analysis. In contrast to HIVdb, COMET and SmartGene^®^, the Rega tool has not been updated for many years and showed to be unsuitable for the identification HIV-1 sub-subtype A6 in our study. Indeed, in its subset of HIV-1 subtype A reference sequences only sub-subtype A1 and A2 sequences are included.

Also, for the identification of RAMs, variations are observed depending on the tool used: HIVdb, ANRS or Cutrell. For RPV, the HIVdb algorithm includes twice as many RAMs as ANRS and Cutrell. However, the additional RAMs identified by HIVdb are rare and typically confer only low-level resistance. In our dataset, the E138A mutation was detected in 6.6% of cases classified as sub-subtype A6 and was consistently identified by all three methods. According to HIVdb, the E138A mutation confers intermediate resistance to RPV, while ANRS classifies it as fully resistant to RPV. Additionally, only the HIVdb algorithm classified G190S (found in 1.5% of sub-subtype A6 cases) as intermediate resistant and V179D (present in 0.7% of sub-subtype A6 cases) as susceptible to RPV. Hence, in case of RPV resistance, the selection of the tool used did not have a huge impact on the number of people eligible to receive long-acting medication in our dataset. When performing the same analysis for CAB, more pronounced differences emerged as the ANRS algorithm only scored half as many RAMs compared to the list of Cutrell and the HIVdb algorithm [8]. However, only half of the RAMs scored by the HIVdb algorithm confer intermediate or high-level resistance to CAB. Analyzing our dataset with the ANRS algorithm did not identify any RAMs, while HIVdb identified the RAMs L74M (2.6%) and Y143C (1.3%), that nevertheless were scored as susceptible. According to the Cutrell list, RAMs against CAB were present in 98.7% of sub-subtype A6 cases, which was primarily due to the scoring of L74I (96.1%) next to G193E (3.9%), L74M (2.6%) and Y143C (1.3%).

As the L74I mutation was observed in the small number of patients who failed LA-CAB + LA-RPV in clinical trials [3,4,36], and it was one of the integrase mutations previously associated with dolutegravir exposure, it was incorporated in the list of mutations potentially associated with resistance to INSTI by Cutrell et al. (2021) [8]. Another study identified L74I as a key genetic marker of HIV-1 sub-subtype A6 [37]. Indeed, it was only found in 7% of 5754 integrase genes from isolates within the LANL HIV sequence database, whereas it was present in 92% of HIV-1 sub-subtype A6 isolates [38]. Cutrell et al. (2021) [8] could not demonstrate a significant association between L74I and confirmed virologic failure despite its strong correlation with HIV-1 sub-subtype A6, one of the markers for failure in the clinical studies. In vitro, the L74I mutation did not impact CAB susceptibility, either within a background subtype B or A6 sequence [38]. Unlike the L74M mutation, which is associated with reduced susceptibility to INSTIs when combined with certain resistance-associated mutations, L74I is most likely not associated with CAB resistance, suggesting that relying on the Cutrell list as it stands could unjustly restrict access to long-acting medication for many individuals, and that HIVdb and ANRS are better alternatives. Additionally, they have the added value that they are integrated in software tools facilitating drug resistance interpretation in clinical practice. These tools are not only more user-friendly but also offer broader applicability by enabling the scoring of resistance across multiple classes of HIV medications. Importantly, their regular updates ensure that they remain aligned with the latest scientific evidence, likely enhancing their reliability and accuracy over time.

In summary, the rise in HIV-1 sub-subtype A6 diagnoses in Belgium since 2020 reflects evolving transmission dynamics, particularly among MSM born in Belgium, and cannot be solely attributed to recent Ukrainian migration. Moreover, we demonstrated that the number of sequences displaying some resistance to LA-CAB + LA-RPV in our sub-subtype A6 Belgian baseline dataset remains very low, with no resistance against CAB and only 11 patients displaying some level of RPV resistance due to E138A and/or G190S within RT. HIVdb and ANRS were highly concordant in estimating drug resistance levels within this therapy-naïve population and HIVdb, COMET and SmartGene^®^ were concordant automatic subtyping tools. While the impact of algorithm selection may be marginal in cases of low resistance prevalence, further studies are needed to evaluate the utility of these tools in other settings. However, the dynamic nature of HIVdb and ANRS makes them preferable for ongoing clinical and research applications.

## Figures and Tables

**Table 1:** Characteristics of HIV-1 individuals diagnosed in Belgium between 2013 and 2022.

Characteristics, n (%)		All Cases	Therapy-Naïve and HIV-1 Sub-Subtyped A6 Cases		
**Phylogenetic cluster patterns**		**Total**	**Singletons/Pair**	**Clusters**	**Total**
		7128 (100.0%)	107 (78.7%)	29 (21.3%)	136 (100.0%)
**Sex, n (%)**					
	Male	5132 (72.1%)	79 (73.8%)	27 (93.1%)	106 (77.9%)
	Female	1984 (27.9%)	28 (26.2%)	2 (6.9%)	30 (22.1%)
	Unknown	12	0	0	0
**Age, years**					
	Median	37	38	31	36
	IQR (Q1–Q3)	29–47	31–49	30–45	31–47
	Unknown	17	0	0	0
**Country of birth, n (%)**					
	Belgium	2909 (47.8%)	20 (27.0%)	17 (77.3%)	37 (38.5%)
	EER	123 (2.0%)	48 (64.9%)	1 (4.5%)	49 (51.0%)
	Other	3059 (50.2%)	6 (8.1%)	4 (18.2%)	10 (10.4%)
	Unknown	1037	33	7	40
**Risk category, n (%)**					
	MSM	2979 (52.1%)	29 (38.2%)	21 (87.5%)	50 (50.0%)
	HET	2567 (44.9%)	37 (48.7%)	3 (12.5%)	40 (40.0%)
	IDU	89 (1.6%)	10 (13.2%)	0 (0.0%)	10 (10.0%)
	Other	80 (1.4%)	0 (0.0%)	0 (0.0%)	0 (0.0%)
	Unknown	1413	31	5	36
**Diagnosis, year**					
	Median	2017	2018	2021	2020
	IQR (Q1–Q3)	2014–2019	2015–2022	2020–2022	2016–2022

n, number; EER, Eastern European Region; MSM, men having sex with men; HET, heterosexual; IDU, intravenous drug use.

**Table 2:** HIV-1 sub-subtype A6 assignments according to different subtyping tools.

Individual Tools		Combined Approach with Phylogeny		Accuracy	Sensitivity	Specificity
		A6	Other A			
HIVdb	A6	136	15	0.96	1.00	0.95
	Other A	0	276			
COMET	A6	134	0	0.99	0.98	1.00
	Other A	2	291			
Rega	A6	0	0	0.68	0.00	1.00
	Other A	136	291			
SmartGene *	A6	136	0	1.00	1.00	1.00
	Other A	0	291			

* 19 sequences were assigned to HIV-1 sub-subtype A6 in protease and another variant within HIV-1 subtype A in reverse transcriptase (potential recombinants).

## Data Availability

The entire dataset for this manuscript is not publicly available because it represents a dense sample of our epidemic and inappropriate use could endanger the privacy of the patients. A proportional down-sampling of the HIV-1 sub-subtype A6 dataset (10%) is accessible via GenBank (accession numbers, PV254986-PV254999). Requests to access the entire dataset combined with well-defined project proposals can be mailed to the corresponding author who will submit it to the local ethical committee for ethical and legal clearance when not in conflict with already ongoing research.

## Supplementary Materials

The following supporting information can be downloaded at: https://www.mdpi.com/article/10.3390/v18050554/s1, Figure S1: Maximum likelihood phylogenetic tree featuring all HIV-1 sequences with evidence of pure subtype A fragments; Figure S2: Maximum likelihood phylogenetic tree featuring all HIV-1 sequences with evidence of pure sub-subtype A6 fragments; Table S1: Rilpivirine mutations in HIV-1 sub-subtype A6 strains isolated in Belgium (2013–2022); Table S2: Cabotegravir mutations in HIV-1 sub-subtype A6 strains isolated in Belgium (2013–2022).

## Abbreviations

The following abbreviations are used in this manuscript:

ARL	AIDS Reference Laboratories
BMI	Body Mass Index
CAB	Cabotegravir
DRC	Democratic Republic of Congo
EER	Easter European Region
FSU	Former Sovjet Union
HET	Heterosexuals
HIV	Human Immunodeficiency Virus
HLR	High Level Resistance
IDU	Intravenous Drug Users
IDNS	Integrated Database Network System
IN	Integrase
INSTI	Integrase Strand Transfer Inhibitor
IR	Intermediate Resistance
LA	Long-acting
LANL	Los Alamos HIV Sequence Database
LLR	Low Level Resistance
MSM	Men who have Sex with Men
NNRTI	Non-Nucleoside Reverse Transcriptase Inhibitor
PLWH	People Living With HIV
PR	Protease
PrEP	Pre-Exposure Prophylaxis
R	Resistance
RAM	Resistance Associated Mutation
RPV	Rilpivirine
RT	Reverse Transcriptase
