# Supplementary material for: Emerging Trends in HIV-1 Sub-Subtype A6 in Belgium: Transmission Dynamics, Drug Resistance, and Subtyping Tool Evaluation
Source: Viruses. 2026 May 12;18(5):554. doi: 10.3390/v18050554 (PMC13211556; doi:10.3390/v18050554)
Supplement: Supplementary file 1 [file viruses-18-00554-s001.zip › viruses-4303118-supplementary/Supplementary Tables and Figures.pdf]

Table S1: Rilpivirine mutations in HIV-1 sub-subtype A6 strains isolated in Belgium (2013-2022).

Mutation	Cutrell	HIVdb	ANRS	Number	Frequency (%)
A98G		LLR	IR		
L100I	√	HLR			
L100V		LLR			
K101E	√	IR	R		
K101H		PLLR			
K101P	√	HLR	R		
V106I		PLLR			
E138A	√	LLR	R	9	6.6%
E138G	√	LLR	R		
E138K	√	IR	R		
E138Q	√	LLR	R		
E138R	√	LLR	R		
E138S			R		
V179D		PLLR		1	0.7%
V179E		PLLR			
V179F		LLR			
V179L	√	LLR	R		
Y181C	√	IR	R		
Y181I	√	HLR	R		
Y181F		IR			
Y181G		IR			
Y181S		IR			
Y181V	√	HLR	R		
Y188F		IR			
Y188L	√	HLR	R		
G190A		LLR			
G190C		PLLR			
G190E		HLR			
G190Q		IR			
G190S		LLR		2	1.5%
G190T		PLLR			
G190V		PLLR			
H221Y	√	LLR	R		
F227C	√	IR	R		
M230I	√	IR	R		
M230L	√	HLR	R		
M230V			R		

The number, frequency and impact of single mutations within HIV-1 RT: NNRTI mutations according to Cutrell et al. (2021), rilpivirine mutations according to HIVdb v9.6 and ANRS v35. √, included in list or algorithm. PLLR, potential low-level resistance; LLR, low-level resistance; IR, intermediate resistance; HLR, high-level resistance; R, resistance. For comparison, resistance interpretation according to 3-level resistance score: susceptible (green), intermediate resistance (yellow) and resistance (red).

Table S2: Cabotegravir mutations in HIV-1 sub-subtype A6 strains isolated in Belgium (2013-2022).

Mutation	Cutrell	HIVdb	ANRS	Number	Frequency (%)
H51Y	√	LLR			
T66A	√				
T66I	√	PLLR			
T66K	√	LLR	IR		
L68I	√				
L68V	√				
L74F		PLLR			
L74I	√			73	96.1%
L74M	√	PLLR		2	2.6%
V75A		PLLR			
E92G	√	PLLR			
E92Q	√	LLR			
E92V	√	PLLR			
Q95K	√				
T97A	√				
G118R	√	HLR	R		
F121C		HLR			
F121Y	√	LLR	R		
T122N		LLR			
E138A	√	LLR	R		
E138D	√				
E138K	√	LLR	R		
E138T	√	LLR	R		
G140A	√	LLR	R		
G140C	√	LLR	R		
G140R	√	HLR	R		
G140S	√	LLR	R		
Y143A	√	PLLR			
Y143C	√	PLLR		1	1.3%
Y143G	√	PLLR			
Y143H	√	PLLR			
Y143K	√	PLLR			
Y143R	√	PLLR			
Y143S	√	PLLR			
N144D			R		
P145S	√				
Q146L		LLR			
Q146P		LLR			
S147G	√	LLR			
Q148H	√	HLR	R		
Q148K	√	HLR	R		
Q148N		PLLR			

Q148R	√	HLR	R		
V151A	√				
V151I	√				
V151L	√	LLR	R		
S153F	√	LLR	R		
S153Y	√	LLR	R		
N155H	√	IR	R		
N155S	√	LLR			
N155T	√	LLR			
E157Q	√				
G163K	√				
G163R	√				
G193E	√			3	3.9%
S230R	√	LLR	R		
R263K	√	HLR	R		

The number, frequency and impact of single mutations within HIV-1 IN: INSTI mutations according to Cutrell et al. (2021), cabotegravir mutations according to HIVdb v9.6 and ANRS v35. √, included in list or algorithm. PLLR, potential low-level resistance; LLR, low-level resistance; IR, intermediate resistance; HLR, high-level resistance; IR, intermediate resistance; R: resistance. For comparison, resistance interpretation according to 3-level resistance score: susceptible (green), intermediate resistance (yellow) and resistance (red).

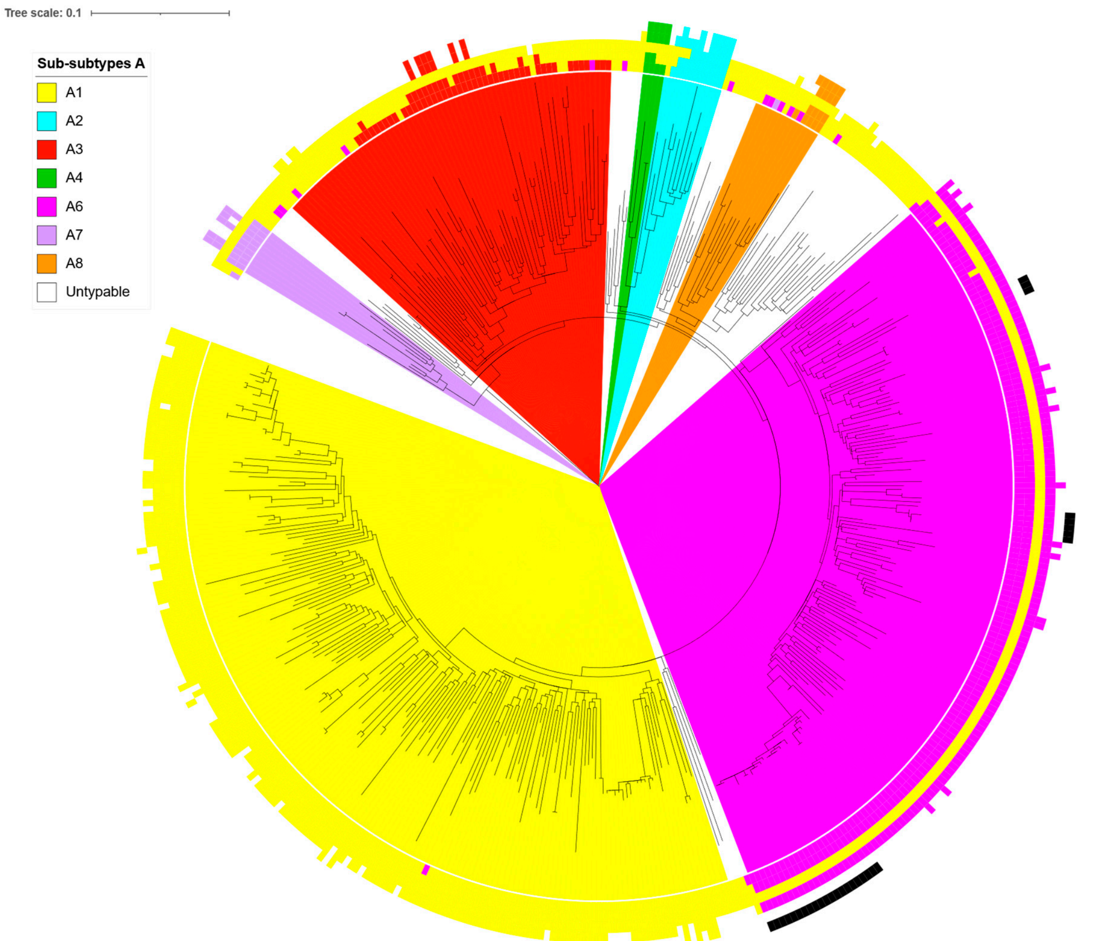

Figure S1: Maximum likelihood phylogenetic tree featuring all HIV-1 sequences with evidence of pure subtype A fragments. Color-coded ranges represent HIV-1 sub-subtype clades with a branch support > 70% (bootstrap analysis using 100 replicates) (selected model GTR +G +I). Layer one subtyping according to HIVdb 9.6, Layer two subtyping according to COMET HIV-1 v2.4, Layer three subtyping according to Rega v3.46, Layer four subtyping according to SmartGene v3_13_0(r32707), Layer five reference sequences from Los Alamos, Layer six clusters identified in the sub-subtype A6 clade (black stripes).

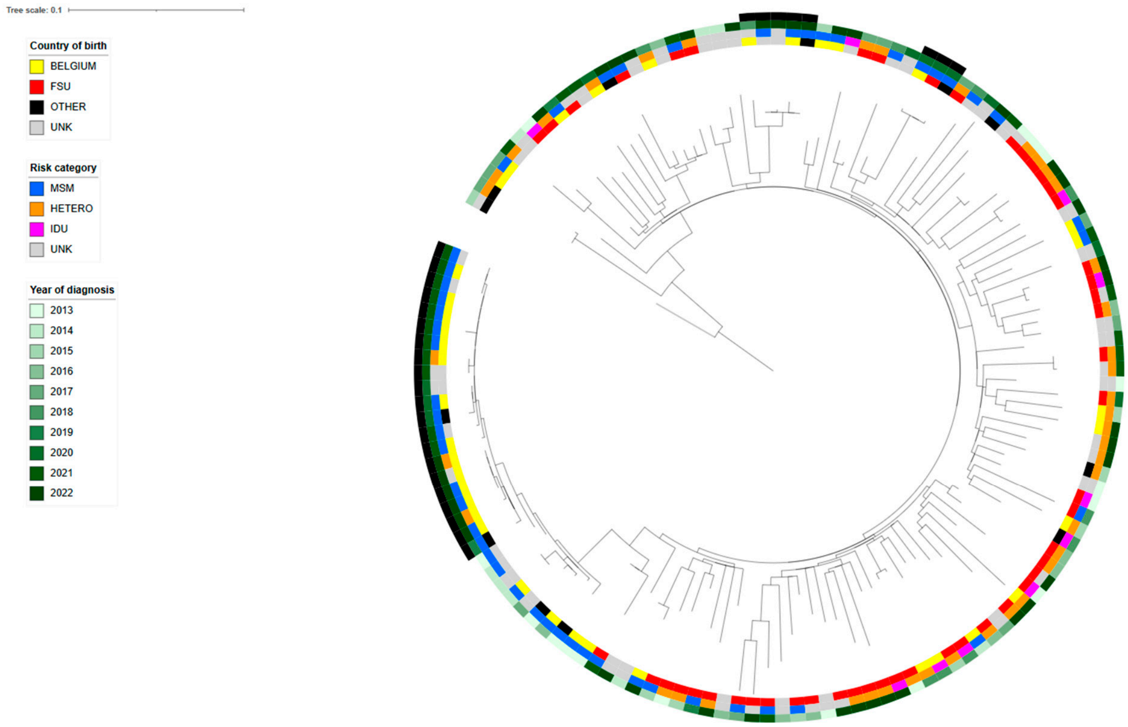

Figure S2: Maximum likelihood phylogenetic tree featuring all HIV-1 sequences with evidence of pure sub-subtype A6 fragments. Layer one annotation based on country of birth, Layer two annotation based on risk category, Layer three annotation based on year of diagnosis, Layer four clusters identified in the sub-subtype A6 clade (black stripes).
